# Global and Regional Trends in Multiple Sclerosis Incidence with Projections to 2030: A Joinpoint Regression Analysis of the GBD Data 2021 by Socio-Demographic Index and WHO Regions

**DOI:** 10.5334/aogh.5438

**Published:** 2026-09-08

**Authors:** Hamed Delam, Naeimehossadat Asmarian, Haleh Ghaem

**Affiliations:** 1 Student Research Committee, Shiraz University of Medical Sciences, Shiraz, Iran; 2 Anesthesiology and Critical Care Research Center, Shiraz University of Medical Sciences, Shiraz, Iran; 3 Research Center for Health Sciences, Institute of Health, Department of Epidemiology, School of Health, Shiraz University of Medical Sciences, Shiraz, Iran

**Keywords:** multiple sclerosis, incidence, forecasting, global burden of disease, epidemiology

## Abstract

*Background:* Multiple sclerosis (MS) constitutes one of the foremost neurological causes of disability among younger and middle-aged populations.

*Objective:* This study aimed to examine global and regional trends in MS incidence from 1990 to 2021, with projections to 2030, using joinpoint regression and back-testing validation to ensure evidence-based scenario selection.

*Methods:* Data were extracted from the Global Burden of Disease (GBD) 2021 study. Joinpoint regression was used to calculate annual percentage changes (APC) and average annual percentage changes (AAPC) for 1990–2021. A retrospective validation (back-testing) was conducted using data from 1990 to 2015 as the training period to project rates for 2016–2021, comparing predictive accuracy using mean absolute percentage error (MAPE) and root mean square error (RMSE). The method with lower error metrics was selected as the primary projection scenario for 2030, with the alternative retained for sensitivity analysis.

*Findings:* Globally, the age-standardized incidence rate (ASIR) decreased slightly from 0.77 to 0.75 per 100,000 (1990–2021), while new cases increased by 49.9%. High socio-demographic index (SDI) (1.66) and EURO (2.57) regions had the highest ASIR in 2021, while low SDI (0.29) and Western Pacific Region (WPRO) (0.15) had the lowest. The back-testing identified APC as the preferred method in 10 of 12 regions (83.3%), with the lowest MAPE in AFRO (0.05%) and the highest discrepancy in South-East Asia Region (SEARO) (APC: 0.36% vs AAPC: 5.45%). Global ASIR is projected to decrease slightly to 0.747 by 2030, while SEARO (+12.3%) and low-middle SDI (+8.2%) regions show the largest increases. Sensitivity analysis confirmed robust projections globally, with trend reversals observed in SEARO and low SDI regions.

*Conclusion:* Global MS incidence is projected to rise in most regions by 2030, particularly in transitioning economies and Southeast Asia. Strengthening diagnostic infrastructure and implementing context-specific health policies are urgently needed.

## Introduction

Multiple sclerosis (MS) is characterized as a chronic autoimmune-mediated disorder of the central nervous system in which the immune response targets the myelin sheath surrounding neuronal axons [1]. This pathological process leads to disruption of nerve signal transmission and a wide range of clinical symptoms, including muscle weakness, visual disturbances, ataxia, chronic fatigue, and cognitive problems [2]. The precise etiology of MS remains incompletely elucidated; however, both hereditary predisposition and environmental exposures are believed to contribute to its pathogenesis [3]. This disease is distributed in a distinct geographic pattern, with its incidence increasing at higher latitudes [4].

In the past three decades, the epidemiological indicators of this disease have changed significantly in most parts of the world, and according to the Atlas of MS, Third Edition, about 2.8 million individuals worldwide are living with this disease, which is equivalent to a prevalence of 35.9 cases per 100,000 population; the cumulative incidence rate among 75 reporting countries is 2.1 cases per 100,000 population per year, and the average age of diagnosis is reported to be 32 years [5]. On the other hand, according to estimates from the Global Burden of Disease (GBD) 2021 Study, more than 62,000 new cases of MS were diagnosed in 2021, and the global prevalence of this disease was 23.9 cases per 100,000 population, showing a continuous increasing trend over the past three decades; regionally, North America has the highest prevalence, and Sweden with 219 cases, Canada with 182 cases, Norway with 176 cases, and the United Kingdom with 158 cases per 100,000 population are among the countries with the highest prevalence [6]. The prevalence and incidence of MS are increasing worldwide, and this increase is likely the result of a combination of improved diagnostic methods, increased awareness, and actual changes in disease incidence [4].

In the Asia-Pacific region, the prevalence of MS is increasing, although the rate is significantly lower than in Western countries [7]. In China, the incidence of this disease has increased in recent years due to the use and expansion of magnetic resonance imaging and new diagnostic criteria [8]. In China, the age-standardized prevalence rate (ASIR) of MS increased from 3.93 per 100,000 population in 1990 to 5.63 per 100,000 by 2019 [9]. According to a systematic review conducted across Europe, the highest prevalence and incidence figures were observed in the Nordic nations and the northern territories of the British Isles [10]. In Spain, the number of cases per 100,000 population has increased by 1.34 every 10 years [11].

MS is the foremost cause of non-traumatic neurological impairment in young adults [12] and, in addition to the human burden of progressive disabilities, imposes a heavy financial burden on health systems, patients, and society [13, 14]. On the one hand, given the increasing number of cases worldwide and the associated rising costs, a detailed understanding of the incidence patterns and time trends of MS is essential for health policymaking and optimal resource allocation; therefore, the present study aimed to estimate the ASIR of MS globally and in different regions based on the socio-demographic index (SDI) and the six regions of the World Health Organization (WHO), examine the time trends of this rate from 1990 to 2021 using the joinpoint regression model, and predict future trends until 2030.

## Method

### Type of study

The present study was an ecological study with a secondary analysis approach of GBD data.

### Research population

The statistical population of this study consisted of all GBD data at the global level for MS from 1990 to 2021. The sampling method in this study was census, meaning that all available and accessible data on the incidence of MS during the period 1990–2021 were extracted from the GBD database. The global data included information on the entire world, the six WHO regions (Africa [AFRO], America [AMRO], Eastern Mediterranean [EMRO], Europe [EURO], Southeast Asia [SEARO], and Western Pacific [WPRO]), as well as regions with different SDI (low, low‐middle, middle, high-middle, and high). All required data were extracted from the GBD Study website at ghdx.healthdata.org/gbd [15] and then used for statistical analyses.

### Study variables

The variables under study were defined as follows to determine the number of new cases, ASIR, time trend, and projection to 2030. The main variable was the ASIR of MS. This variable was defined as the number of new cases of the disease per 100,000 population in a given year. Age standardization was performed using the direct method and based on the global standard population, which was calculated and published by the Institute for Health Metrics and Evaluation (IHME) in the GBD study. In addition to the ASIR, the number of new cases of MS (Count) was also used as another variable. The ASIR and the number of new cases of the disease were reported by sex and year in the study areas.

### Temporal change indices

In this study, two main indices were calculated to quantify the trend in the ASIR of MS over time, including the annual percentage change (APC) and the average annual percentage change (AAPC). These indices were obtained for each of the regions studied worldwide (including SDI regions and WHO regions). The significance level in this study was considered to be 0.05.

### Projections of ASIR to 2030

The ASIRs of MS to 2030 were predicted using the APC and AAPC methods. These projections were applied to all regions studied globally (SDI regions and WHO regions). In the APC method, the trend observed in the last time slot identified by the joinpoint model was assumed to continue until 2030. In other words, the APC calculated for the last time slot (representing the current and recent trend of the disease) was taken as the annual growth rate for the period 2021–2030. This method better reflects recent changes and was more suitable for regions whose trends changed in the final years of the study. The AAPC method assumed that the average long-term trend for the entire period 1990–2021 would continue through 2030 [16]. The AAPC, which represents the average annual growth or decline over the 32-year period, was taken as the annual growth rate over the prediction period. This method provides a more general picture of the historical trend and adjusts for short-term fluctuations. For both prediction methods, two main indicators were calculated to quantify the changes between 2021 and 2030.

The projections of ASIR to 2030 were calculated using the following formulas:


$${A\,S\,I\,R}_{A\,P\,C}\,\left(t\right)=A\,S\,I\,R\left(2021\right)\times {\left(1+\frac{{A\,P\,C}_{fi\,n\,a\,l}}{100}\right)}^{t-2021}$$

$${A\,S\,I\,R}_{A\,A\,P\,C}\,\left(t\right)=A\,S\,I\,R\left(2021\right)\times {\left(1+\frac{A\,A\,P\,C}{100}\right)}^{t-2021}$$

where:

*APC*_f*inal*_ = Annual percentage change for the last identified segment in the joinpoint model

*AAPC* = Average annual percentage change for the entire 1990–2021 period

*t* = target year (2022–2030).

### Back-Testing validation

To evaluate the predictive accuracy of the APC and AAPC projection methods, a retrospective validation (back-testing) procedure was conducted. This approach involves fitting the joinpoint regression model on a historical subset of the observed data and comparing the projected values against the actual observed data for a subsequent validation period.

#### Validation framework

The back-testing procedure was implemented using a rolling window approach, in which the joinpoint model was fitted to data from 1990 to 2015 (training period) to project ASIRs for 2016–2021 (validation period). The projected values were then compared with the actual observed GBD data for 2016–2021 using established error metrics. The choice of the validation period (2016–2021) ensured that the models were tested on recent data, while the training period (1990–2015) provided sufficient historical data to reliably estimate the joinpoint segments and APC values. This approach allows for the empirical assessment of which projection method APC or AAPC more accurately captures future epidemiological patterns.

#### Error metrics

Two standard forecast accuracy metrics were employed to compare the predictive performance of the APC and AAPC methods [17]:

*Mean absolute percentage error (MAPE):*


$$MAPE=\frac{1}{n}\sum_{2016}^{2021}|\frac{{ASIR}_{real}\left(t\right)\;-{ASIR}_{predict}(t)}{{ASIR}_{real}(t)}|\times 100$$

*Root mean square error (RMSE):*


$$RMSE=\sqrt{\frac{1}{n}\sum_{2016}^{2021}({ASIR}_{real}\left(t\right)-{ASIR}_{predict}(t){)}^{2}}$$

where:

ASIR_real_ (*t*) = the observed ASIR in year *t*

ASIR_predict_ (*t*) = the projected ASIR in year *t*

*n* = the number of years in the validation period (2016–2021; *n* = 6).

MAPE provides a percentage-based measure of forecast accuracy, while RMSE captures the magnitude of prediction errors in the original units (per 100,000 population) and is more sensitive to large errors [18].

#### Method selection

Based on the back-testing results, the method with lower MAPE and RMSE for each region was selected as the primary projection method for the 2030 forecasts. The alternative method was retained as a sensitivity scenario to assess the robustness of the projections.

### Sensitivity analysis

Following the back-testing validation, the alternative projection method (the one not selected as primary for each region) was retained as a sensitivity scenario to assess the robustness of the 2030 projections. This approach allowed us to evaluate the stability of the forecasts by comparing the range of estimates produced under different trend assumptions.

#### Sensitivity indicators

The following indicators were calculated to quantify the sensitivity of the projections [19]:

Sensitivity Index: The absolute difference between the primary and alternative scenario projections for 2030:

Sensitivity Index = |Primary Projection 2030 − Alternative Projection 2030| (per 100,000 population)

Relative Change: The percentage difference between the primary and alternative scenario projections:

Relative Change (%) = (Sensitivity Index / Primary Projection 2030) × 100

Trend reversal defined as opposite directions of change between primary and alternative scenarios (one increasing, the other decreasing).

### Statistical software

All joinpoint regression analyses were performed using the Joinpoint Regression Program (version 5.3.0.0, National Cancer Institute) [20]. The model was specified as a log-linear regression of the ASIR. Since joinpoint regression is a standard and validated method for analyzing longitudinal trends in epidemiology, especially for incidence and mortality rate data, its use in the GBD study data is statistically justified [20]. The method was developed by the National Cancer Institute (NCI) and has been widely used in analyzing trends in cancer and noncommunicable diseases [20, 21]. To account for the inherent uncertainty and heteroscedasticity of the GBD estimates, the Standard Error (Provided) option was selected. This performs a weighted least squares (WLS) regression, using the standard errors of the age-standardized rates as weights to stabilize variance and ensure robust parameter estimation [22]. The optimal number of joinpoints was determined using the Weighted Bayesian Information Criterion (Weighted BIC), which is the default and recommended method in Joinpoint version 5.0 and above. The maximum number of joinpoints was set to 6, with a minimum of 5001 resamples for model selection. The minimum number of observations between joinpoints was set according to the software’s default recommendations.

From a methodological perspective, the joinpoint model is designed to identify significant change points in time trends and estimate the APC and AAPC, which average out and attenuate random fluctuations [20]. In addition, by limiting the number of joinpoints and using log-linear models on age-standardized rates, overfitting of relatively volatile data can be avoided [20, 21].

All graphical presentations and additional statistical analyses were performed using GraphPad Prism 10 and Microsoft Excel.

## Results

### ASIR estimates

#### Global ASIR

Globally, the ASIR of MS decreased slightly from 0.77 (95% CI: 0.68–0.86) per 100,000 population in 1990 to 0.75 (95% CI: 0.68–0.82) in 2021. This decreasing trend was observed in both sexes, with a more pronounced decline among males (from 0.52 to 0.50) compared to females (from 1.02 to 1.01). The female-to-male ratio remained relatively stable over the study period.

#### ASIR by SDI regions

In 2021, the highest ASIR among SDI regions was observed in the high SDI region (1.66 per 100,000), while the lowest ASIR was found in the low SDI region (0.29 per 100,000). The largest increase from 1990 to 2021 occurred in the high SDI region (+0.29), whereas the low and low-middle SDI regions remained relatively stable (Table 1).

**Table 1:** Number of new cases and ASIR of multiple sclerosis in global and by SDI and WHO regions.

LOCATION	NUMBER (95% CI)						ASIR PER 100,000 (95% CI)					
	SEX						SEX					
	BOTH		MALE		FEMALE		BOTH		MALE		FEMALE	
	1990	2021	1990	2021	1990	2021	1990	2021	1990	2021	1990	2021
**Global**	41,970 (36,605–48,234)	62,920 (56,015–70,635)	15,801 (13,755–18,219)	23,728 (20,908–26,691)	26,169 (22,883–29,999)	39,192 (35,032–44,052)	0.77 (0.68–0.86)	0.75 (0.68–0.82)	0.52 (0.47–0.58)	0.50 (0.45–0.55)	1.02 (0.90–1.13)	1.01 (0.92–1.10)
**SDI Regions**												
Low	1456 (1184–1792)	3974 (3311–4754)	555 (450–686)	1468 (1209–1782)	901 (735–1102)	2506 (2086–2966)	0.30 (0.25–0.34)	0.29 (0.25–0.34)	0.23 (0.19–0.26)	0.22 (0.18–0.25)	0.38 (0.31–0.43)	0.37 (0.31–0.43)
Low-middle	4107 (3358–4995)	9858 (8341–11,614)	1625 (1331–1994)	3702 (3060–4407)	2483 (2039–3001)	6156 (5205–7238)	0.34 (0.29–0.39)	0.34 (0.29–0.39)	0.24 (0.20–0.27)	0.23 (0.19–0.26)	0.45 (0.38–0.52)	0.45 (0.38–0.51)
Middle	6146 (5141–7333)	12,594 (10,823–14,531)	2450 (2048–2936)	5094 (4334–5844)	3696 (3073–4407)	7500 (6423–8701)	0.30 (0.26–0.35)	0.37 (0.31–0.42)	0.20 (0.17–0.23)	0.23 (0.19–0.26)	0.41 (0.35–0.47)	0.51 (0.43–0.59)
High-middle	9143 (8123–10,318)	10,507 (9447–11,562)	3812 (3366–4309)	4475 (3998–4943)	5331 (4746–5998)	6032 (5443–6677)	0.30 (0.26–0.34)	0.39 (0.34–0.43)	0.20 (0.17–0.23)	0.25 (0.22–0.28)	0.41 (0.35–0.46)	0.53 (0.46–0.59)
High	21,063 (18,609–23,755)	25,926 (23,686–28,293)	7337 (6481–8301)	8966 (8180–9808)	13,726 (12,198–15,549)	16,961 (15,492–18,534)	1.37 (1.23–1.52)	1.66 (1.55–1.79)	0.93 (0.83–1.02)	1.07 (0.99–1.16)	1.83 (1.63–2.02)	2.30 (2.15–2.47)
**WHO Regions**												
AFRO	1239 (1028–1458)	3114 (2591–3628)	449 (372–531)	1094 (910–1282)	790 (660–933)	2020 (1682–2352)	0.31 (0.26–0.35)	0.31 (0.27–0.36)	0.24 (0.20–0.27)	0.24 (0.20–0.27)	0.38 (0.31–0.43)	0.39 (0.33–0.44)
AMRO	11,005 (9636–12,383)	16,905 (15,826–18,105)	3399 (2999–3783)	5457 (5052–5913)	7606 (6667–8591)	11,447 (10,757–12,258)	1.53 (1.34–1.72)	1.60 (1.50–1.71)	0.99 (0.87–1.10)	1.03 (0.96–1.11)	2.05 (1.80–2.32)	2.16 (2.03–2.31)
EMRO	3193 (2714–3736)	7712 (6704–8670)	992 (841–1150)	2447 (2123–2744)	2201 (1875–2582)	5265 (4568–5926)	0.95 (0.81–1.08)	1.04 (0.91–1.17)	0.58 (0.50–0.66)	0.64 (0.56–0.72)	1.34 (1.13–1.53)	1.49 (1.29–1.68)
EURO	18,670 (16,851–20,566)	24,534 (22,433–26,760)	6508 (5832–7177)	8401 (7619–9203)	12,161 (11,003–13,361)	16,133 (14,741–17,591)	2.07 (1.88–2.27)	2.57 (2.38–2.78)	1.48 (1.33–1.62)	1.74 (1.60–1.89)	2.66 (2.42–2.92)	3.41 (3.16–3.68)
SEARO	2885 (2390–3407)	5047 (4178–5844)	1052 (865–1250)	1800 (1487–2112)	1833 (1525–2158)	3247 (2703–3725)	0.28 (0.23–0.32)	0.27 (0.22–0.31)	0.20 (0.16–0.23)	0.19 (0.16–0.22)	0.36 (0.30–0.42)	0.35 (0.29–0.40)
WPRO	2184 (1868–2496)	3477 (2961–3946)	701 (589–819)	1158 (979–1332)	1483 (1268–1687)	2319 (2961–3946)	0.13 (0.11–0.15)	0.15 (0.13–0.17)	0.08 (0.07–0.09)	0.10 (0.08–0.11)	0.18 (0.15–0.20)	0.20 (0.18–0.23)

ASIR: Age-standardized incidence rate (per 100,000 population); SDI: Socio-demographic index; AFRO: African region; AMRO: Region of the Americas; SEARO: South-East Asia region; EURO: European region; EMRO: Eastern Mediterranean region; WPRO: Western Pacific region.

#### ASIR by WHO regions

Among WHO regions, the highest ASIR in 2021 was in the European Region (EURO) with 2.57 per 100,000, which was also the region with the largest increase from 1990 (+0.50). The lowest ASIR was in the Western Pacific Region (WPRO) with 0.15 per 100,000 (Table 1).

### Temporal trend of ASIR based on joinpoint regression

#### Global temporal trend

According to Table 2, the global AAPC for 1990–2021 was −0.08%, indicating a very mild decline. Five distinct segments were identified: a significant decline in 1990–1993 (APC: −0.66%, *P* < 0.001), a non-significant plateau in 1993–1996 (APC: −0.32%, *P* = 0.113), a significant increase in 1996–2004 (APC: 0.36%, *P* = 0.022), a significant decline in 2004–2012 (APC: −0.19%, *P* = 0.003), and a mild but significant decline in 2012–2021 (APC: −0.08%, *P* = 0.019).

**Table 2:** Temporal trend analysis in incidence rate of multiple sclerosis in global and by SDI and WHO regions (1990–2021).

LOCATION	AAPC (%) 1990–2021 (95% CI)	SEGMENT(S)		APC (95% CI)	*P*-VALUE
		NUMBER	YEAR		
**Global**	−0.08 (−0.09, −0.07)	5	1990–1993	−0.66 (−0.83, −0.55)	<0.001
			1993–1996	−0.32 (−0.40, 0.28)	0.113
			1996–2004	0.36 (0.33, 0.39)	0.022
			2004–2012	−0.19 (−0.26, −0.16)	0.003
			2012–2021	−0.08 (−0.11, −0.03)	0.019
**Low SDI**	−0.08 (−0.09, −0.07)	3	1990–2005	−0.36 (−0.38, −0.33)	<0.001
			2005–2010	−0.61 (−0.80, −0.52)	0.001
			2010–2021	0.55 (0.52, 0.59)	<0.001
**Low-middle SDI**	−0.04 (−0.04, −0.03)	5	1990–2010	−0.31 (−0.32, −0.30)	<0.001
			2010–2013	0.47 (−0.31, 0.52)	0.099
			2013–2016	0.11 (0.04, 0.45)	0.004
			2016–2019	0.50 (0.15, 0.57)	<0.001
			2019–2021	0.93 (0.77, 1.04)	<0.001
**Middle SDI**	0.63 (0.62, 0.63)	6	1990–1993	−0.55 (−0.60, −0.49)	<0.001
			1993–1996	0.19 (0.14, 0.25)	0.003
			1996–2001	0.62 (0.57, 0.66)	<0.001
			2001–2008	0.79 (0.75, 0.82)	<0.001
			2008–2019	0.89 (0.85, 0.90)	<0.001
			2019–2021	1.04 (0.91, 1.10)	<0.001
**Middle-high SDI**	0.82 (0.81, 0.83)	4	1990–1995	−0.49 (−0.56, −0.42)	<0.001
			1995–2003	1.44 (1.40, 1.51)	0.002
			2003–2009	1.20 (1.06, 1.29)	<0.001
			2009–2021	0.76 (0.73, 0.78)	<0.001
**High SDI**	−0.03 (−0.04, −0.03)	6	1990–1992	−0.84 (−0.94, −0.74)	<0.001
			1992–1995	−0.50 (−0.56, −0.44)	0.005
			1995–1999	0.31 (0.27, 0.37)	<0.001
			1999–2004	0.15 (0.10, 0.19)	0.015
			2004–2007	−0.08 (−0.12, 0.00)	0.060
			2007–2021	0.03 (0.02, 0.05)	0.011
**AFRO**	0.06 (0.05, 0.07)	2	1990–2004	−0.03 (−0.04, −0.02)	<0.001
			2004–2021	0.13 (0.12, 0.14)	<0.001
**AMRO**	0.16 (0.15, 0.17)	6	1990–1992	1.14 (1.03, 1.25)	<0.001
			1992–1995	0.37 (0.30, 0.43)	<0.001
			1995–2000	−0.17 (−0.22, −0.13)	<0.001
			2000–2004	0.24 (0.18, 0.31)	<0.001
			2004–2011	−0.13 (−0.18, −0.11)	<0.001
			2011–2015	0.07 (−0.02, 0.18)	0.106
			2015–2021	0.34 (0.30, 0.39)	<0.001
**EMRO**	0.31 (0.30, 0.33)	4	1990–1992	−1.77 (−2.04, −1.38)	<0.001
			1992–1995	−1.12 (−1.22, 0.88)	0.073
			1995–2010	0.88 (0.84, 0.90)	<0.001
			2010–2021	0.32 (0.29, 0.36)	0.001
**EURO**	0.70 (0.69, 0.71)	7	1990–1993	−0.07 (−0.28, 0.03)	0.142
			1993–1996	0.55 (0.45, 0.64)	<0.001
			1996–1999	2.18 (2.08, 2.26)	<0.001
			1999–2004	1.42 (1.37, 1.47)	<0.001
			2004–2008	0.60 (0.53, 0.81)	<0.001
			2008–2014	0.44 (0.32, 0.49)	<0.001
			2014–2021	0.21 (0.15, 0.25)	0.004
**SEARO**	−0.12 (−0.14, −0.12)	4	1990–2005	−0.58 (−0.59, −0.56)	<0.001
			2005–2010	−0.90 (−0.97, −0.83)	<0.001
			2010–2019	0.76 (0.72, 0.78)	<0.001
			2019–2021	1.27 (1.02, 1.42)	<0.001
**WPRO**	0.50 (0.48, 0.51)	5	1990–1995	−0.27 (−0.48, −0.15)	0.001
			1995–2001	0.80 (0.59, 0.94)	<0.001
			2001–2005	1.45 (1.28, 1.69)	0.001
			2005–2010	−0.43 (−0.57, −0.33)	0.001
			2010–2021	0.76 (0.72, 0.80)	<0.001

APC: Annual percent change; AAPC: Average annual percent change; SDI: Socio-demographic index; AFRO: African region; AMRO: Region of the Americas; SEARO: South-East Asia region; EURO: European region; EMRO: Eastern Mediterranean region; WPRO: Western Pacific region.

#### Temporal trend by SDI regions

In the low SDI region, the overall AAPC was −0.08%, indicating a slight decline; however, a complete trend reversal occurred in the most recent period (2010–2021), with a significant positive APC of 0.55% (*P* < 0.001). The low-middle SDI region showed a very slight decrease (AAPC: −0.04%). In contrast, both the middle SDI (AAPC: 0.63%) and high-middle SDI (AAPC: 0.82%) regions demonstrated strong and significant upward trends, with the high-middle SDI region showing the strongest increase among all SDI regions.

#### Temporal trend by WHO regions

Among WHO regions, EURO had the highest increase (AAPC: 0.70%), with a sharp peak in 1996–1999 (APC: 2.18%) before slowing to 0.21% in 2014–2021. AFRO (0.06%), AMRO (0.16%), and EMRO (0.31%) showed mild to moderate increases. SEARO was the only region with an overall decline (AAPC: −0.12%), but reversed sharply in 2019–2021 (APC: 1.27%, *P* < 0.001). WPRO showed a moderate increase (AAPC: 0.50%) (Table 2).

### Projected ASIR up to 2030

#### Global projected ASIR

**Figure 1:**
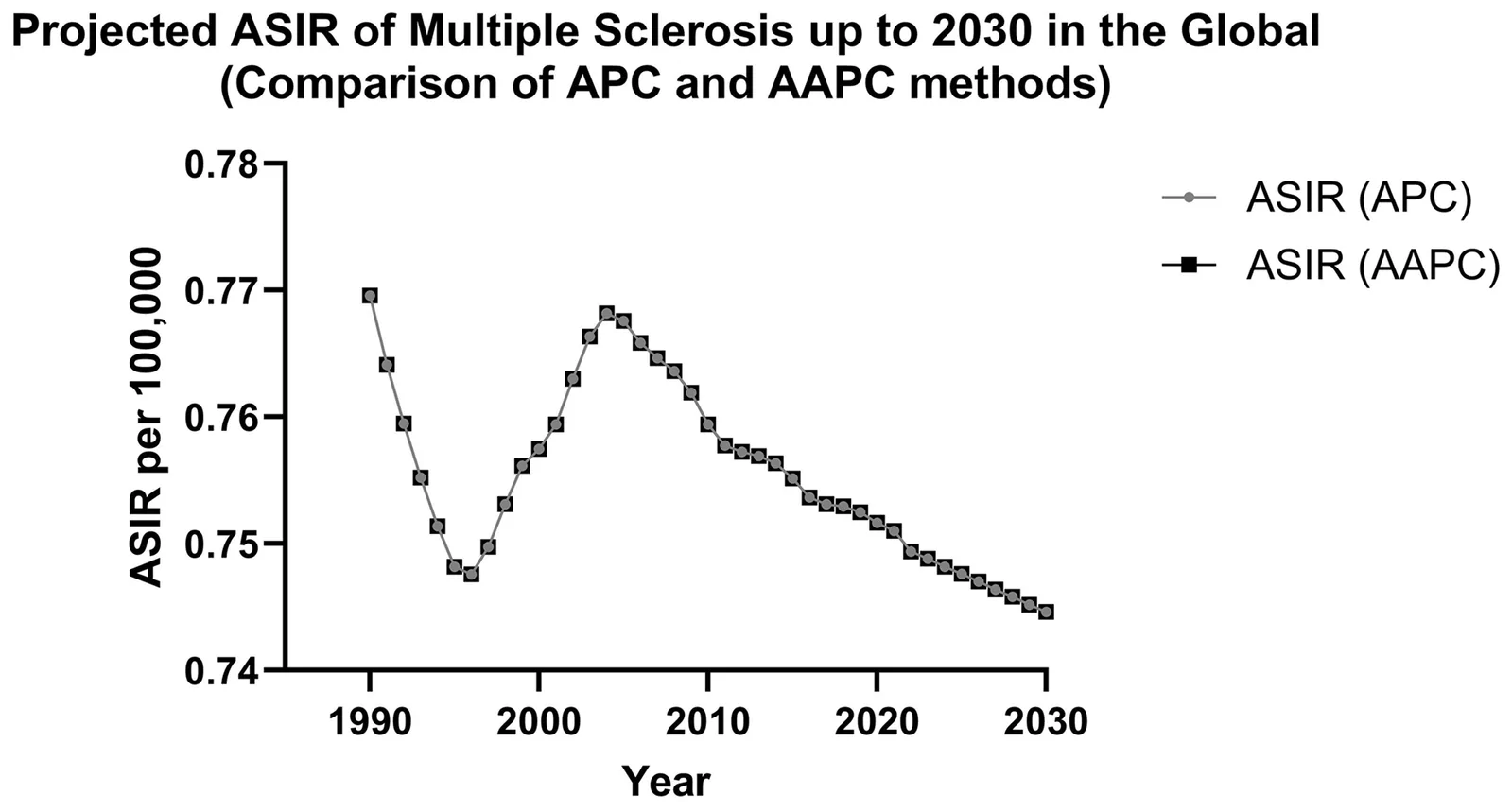
Projected global age-standardized incidence rate (ASIR) of multiple sclerosis up to 2030 using APC and AAPC methods.

According to Table 3, the global ASIR is projected to slightly decrease from 0.753 in 2021 to 0.747 in 2030, with both APC and AAPC methods producing identical projections (absolute change: −0.006, relative change: −0.8%). This indicates a stable and consistent global trend with minimal change over the projection period (Figure 1).

**Table 3:** Projected age-standardized incidence rate (ASIR) of multiple sclerosis in 2030 with absolute and relative changes by region.

REGION	ASIR 2021	APC (%) (LAST SEGMENT)	APC PROJECTION 2030	ABSOLUTE CHANGE (APC)*	RELATIVE CHANGE (APC)**	AAPC (%) (1990–2021)	AAPC PROJECTION 2030	ABSOLUTE CHANGE (AAPC)*	RELATIVE CHANGE (AAPC)**
**Global**	0.753	−0.08	0.747	−0.006	−0.8	−0.08	0.747	−0.006	−0.8
**SDI Regions**									
Low	0.294	+0.55	0.308	+0.014	+4.8	−0.08	0.293	−0.001	−0.3
Low-middle	0.339	+0.93	0.368	+0.029	+8.6	−0.04	0.338	−0.001	−0.3
Middle	0.369	+1.04	0.405	+0.036	+9.8	+0.63	0.390	+0.021	+5.7
High-middle	0.386	+0.76	0.413	+0.027	+7.0	+0.82	0.415	+0.029	+7.5
High	2.370	+0.03	2.376	+0.006	+0.3	−0.03	2.364	−0.006	−0.3
**WHO Regions**									
AFRO	0.314	+0.13	0.318	+0.004	+1.3	+0.06	0.316	+0.002	+0.6
AMRO	1.602	+0.34	1.652	+0.050	+3.1	+0.16	1.625	+0.023	+1.4
EMRO	1.044	+0.32	1.074	+0.030	+2.9	+0.31	1.074	+0.030	+2.9
EURO	2.568	+0.21	2.617	+0.049	+1.9	+0.70	2.735	+0.167	+6.5
SEARO	0.268	+1.27	0.301	+0.033	+12.3	−0.12	0.265	−0.003	−1.1
WPRO	0.149	+0.76	0.160	+0.011	+7.4	+0.50	0.156	+0.007	+4.7

APC: Annual percentage change; AAPC: Average annual percentage change; SDI: Socio-demographic index; WHO: World Health Organization; AFRO: African region; AMRO: Region of the Americas; EMRO: Eastern Mediterranean region; EURO: European region; SEARO: South-East Asia region; WPRO: Western Pacific region.

*Absolute Change = Projected ASIR 2030 − ASIR 2021 (per 100,000 population); **Relative Change (%) = [(Projected ASIR 2030 − ASIR 2021) / ASIR 2021] × 100.

#### Projected ASIR by SDI regions

Projections for SDI regions show divergent patterns. The low SDI region is projected to increase from 0.294 to 0.308 (relative change: +4.8%) using the APC method, while the AAPC method projects a slight decrease (−0.3%). The low-middle SDI region shows a similar discrepancy, with the APC method projecting an increase of +8.6% compared to a slight decrease of −0.3% with the AAPC method. The middle SDI and high-middle SDI regions both show consistent increasing trends across both methods, with relative changes of +5.7%–+9.8% and +7.0%–+7.5%, respectively. The high SDI region remains relatively stable, with a minimal increase of +0.3% under the APC method and a slight decrease of −0.3% under the AAPC method (Table 3).

#### Projected ASIR by WHO regions

Among WHO regions, the European Region (EURO) shows the largest absolute increase, rising from 2.568 in 2021 to 2.735 in 2030 under the AAPC method (relative change: +6.5%). The AMRO region shows a moderate increase from 1.602 to 1.652 (relative change: +3.1%), while the EMRO region shows consistent projections across both methods (relative change: +2.9%). The SEARO region exhibits the largest relative increase (+12.3%) under the APC method, rising from 0.268 to 0.301. However, the AAPC method projects a slight decrease (−1.1%), indicating a substantial discrepancy between short-term and long-term trends. The WPRO region shows an increase from 0.149 to 0.160 (relative change: +7.4%), while the AFRO region remains relatively stable with a mild increase of +1.3% (Table 3).

### Back-Testing validation

#### Global level

According to Table 4, the back-testing validation revealed that the AAPC method demonstrated superior predictive accuracy at the global level, with a MAPE of 0.09% compared to 0.30% for the APC method. The AAPC method also showed a closer projection to the actual 2021 ASIR (0.752 vs. 0.748). Therefore, the AAPC method was selected as the preferred method for global projections.

**Table 4:** Back-testing results comparing predictive accuracy of short-term (APC) versus long-term (AAPC) trend methods for multiple sclerosis age-standardized incidence rates.

REGION	FINAL APC (1990–2015)	AAPC (1990–2015)	ASIR 2021 (ACTUAL)	APC PROJECTION 2021	AAPC PROJECTION 2021	MAPE (APC)	MAPE (AAPC)	PREFERRED METHOD
**Global**	−0.16%	−0.08%	0.753	0.748	0.752	0.30%	0.09%	AAPC
**SDI Regions**								
Low	+0.58%	−0.23%	0.294	0.294	0.280	0.33%	3.04%	APC
Low-middle	+0.12%	−0.19%	0.339	0.329	0.324	0.47%	3.37%	APC
Middle	+0.89%	+0.55%	0.369	0.368	0.360	0.11%	1.53%	APC
High-middle	+0.78%	+0.83%	0.386	0.387	0.389	0.13%	0.59%	APC
High	0.00%	−0.05%	2.370	2.367	2.361	0.11%	0.27%	APC
**WHO Regions**								
AFRO	+0.14%	+0.05%	0.314	0.314	0.311	0.05%	0.34%	APC
AMRO	+0.08%	+0.10%	1.602	1.579	1.581	0.14%	0.60%	APC
EMRO	+0.29%	+0.31%	1.044	1.038	1.040	0.23%	1.77%	APC
EURO	+0.49%	+0.83%	2.568	2.553	2.570	0.29%	0.18%	AAPC
SEARO	+0.77%	−0.38%	0.268	0.264	0.249	0.36%	5.45%	APC
WPRO	+0.80%	+0.44%	0.149	0.148	0.147	0.19%	1.31%	APC

APC: Annual percentage change; AAPC: Average annual percentage change; MAPE: Mean absolute percentage error; RMSE: Root mean square error; SDI: Socio-demographic index; WHO: World Health Organization; AFRO: African region; AMRO: Region of the Americas; EMRO: Eastern Mediterranean region; EURO: European region; SEARO: South-East Asia region; WPRO: Western Pacific region.

#### SDI regions

Across SDI regions, the APC method was identified as the preferred approach in all five SDI regions, consistently showing lower MAPE values compared to the AAPC method. The largest difference was observed in the low-middle SDI region, where the APC method achieved a MAPE of 0.47% versus 3.37% for the AAPC method. The smallest difference was in the high-middle SDI region (0.13% vs. 0.59%). Notably, in the high SDI region, the APC method showed near-perfect accuracy with a MAPE of only 0.11% (Table 4).

#### WHO regions

Among WHO regions, the APC method was the preferred approach in five out of six regions (AFRO, AMRO, EMRO, SEARO, and WPRO). The highest accuracy was observed in AFRO, where the APC method achieved the lowest MAPE among all regions (0.05%). The largest discrepancy between methods was in SEARO, where the APC method (MAPE: 0.36%) substantially outperformed the AAPC method (MAPE: 5.45%), reflecting the divergent short-term and long-term trends in this region. The only exception was EURO, where the AAPC method showed better predictive accuracy (MAPE: 0.18% vs. 0.29% for APC), indicating that long-term historical trends better captured the recent incidence patterns in this region (Table 4).

### Sensitivity analysis

#### Global sensitivity analysis

According to Table 5, at the global level, both primary and alternative scenarios produced identical projections for 2030 (0.747 per 100,000 population), resulting in a sensitivity index of zero and a relative change of 0.0%. No trend reversal was observed, indicating that the global projection is highly robust and independent of the chosen method.

**Table 5:** Primary and alternative scenarios for multiple sclerosis ASIR in 2030 based on back-testing validation.

REGION	ASIR 2021	PRIMARY SCENARIO	PRIMARY PROJECTION 2030	ALTERNATIVE PROJECTION 2030	SENSITIVITY INDEX*	RELATIVE CHANGE (%)**	TREND REVERSAL***
**Global**	0.753	AAPC	0.747	0.747	0.000	0.0	No
**SDI Regions**							
Low	0.294	APC	0.308	0.293	0.015	4.9	Yes
Low-middle	0.339	APC	0.368	0.338	0.030	8.2	Yes
Middle	0.369	APC	0.405	0.390	0.015	3.7	No
High-middle	0.386	APC	0.413	0.415	0.002	0.5	No
High	2.370	APC	2.376	2.364	0.012	0.5	No
**WHO Regions**							
AFRO	0.314	APC	0.318	0.316	0.002	0.6	No
AMRO	1.602	APC	1.652	1.625	0.027	1.6	No
EMRO	1.044	AAPC	1.074	1.074	0.000	0.0	No
EURO	2.568	AAPC	2.735	2.617	0.118	4.3	No
SEARO	0.268	APC	0.301	0.265	0.036	12.0	Yes
WPRO	0.149	APC	0.160	0.156	0.004	2.5	No

APC: Annual percentage change; AAPC: Average annual percentage change; SDI: Socio-demographic index; WHO: World Health Organization; AFRO: African region; AMRO: Region of the Americas; EMRO: Eastern Mediterranean region; EURO: European region; SEARO: South-East Asia region; WPRO: Western Pacific region.

#### Sensitivity analysis by SDI regions

Across SDI regions, the sensitivity analysis revealed varying degrees of uncertainty. The low-middle SDI region showed the largest sensitivity among SDI regions, with a sensitivity index of 0.030 and a relative change of 8.2%, reflecting substantial divergence between the APC-based primary projection (0.368) and the AAPC-based alternative projection (0.338). The low SDI and middle SDI regions also showed moderate sensitivity (relative changes: 4.9% and 3.7%, respectively). In contrast, the high-middle SDI and high SDI regions demonstrated high robustness, with minimal relative changes of 0.5% for both regions. Trend reversals were observed in the low SDI and low-middle SDI regions, where the primary scenario (APC) projected increasing trends while the alternative scenario (AAPC) projected decreasing trends. No trend reversals were observed in the middle SDI, high-middle SDI, or high SDI regions (Table 5).

#### Sensitivity analysis by WHO regions

Among WHO regions, the EMRO region showed the highest robustness, with a sensitivity index of 0.000 and a relative change of 0.0%, indicating identical projections from both scenarios. The AFRO region also demonstrated high stability (relative change: 0.6%), followed by AMRO (1.6%) and WPRO (2.5%). The EURO region showed the largest absolute sensitivity among all WHO regions (sensitivity index: 0.118, relative change: 4.3%), reflecting the substantial difference between the AAPC-based primary projection (2.735) and the APC-based alternative projection (2.617). The SEARO region showed the highest relative change among all regions (12.0%), with a sensitivity index of 0.036, driven by the sharp divergence between short-term and long-term trends. A trend reversal was observed only in the SEARO region, where the primary scenario (APC) projected an increasing trend while the alternative scenario (AAPC) projected a decreasing trend (Table 5).

## Discussion

### Interpretation and comparison of findings

This study examined global and regional trends in the ASIR of MS from 1990 to 2021 and provided projections to 2030. The findings of this study showed that the number of new cases of MS globally increased from 41,970 in 1990 to 62,920 in 2021, an increase of 49.9%, while the ASIR remained almost unchanged during this period, from 0.77 to 0.75 per 100,000 population. This pattern of increasing new cases, coupled with stability in the ASIR, has also been reported in other studies based on global burden of disease data. In a study by Liu et al. [23], who conducted a systematic analysis of 2795 patients from 204 countries and regions, a 49.9% increase in incidence and an 87.9% increase in prevalence were reported, while the ASIR decreased by −3.5% and the age-standardized prevalence decreased by −0.4%, indicating that population growth is the main driver of the increase in absolute cases of the disease globally.

The ASIR in the high SDI region (2.37 per 100,000) was more than six times higher than in the low SDI region (0.29 per 100,000), highlighting a substantial disparity in MS ASIR across the socioeconomic development spectrum. This distribution pattern is closely related to economic and social factors and the level of development of countries. Per capita income, education level, and access to health services are among the most important factors that can affect both the rate of diagnosis and registration of the disease and the actual risk of infection. In the study by Moghaddam et al. [24], which examined the socioeconomic determinants of the global distribution of MS, it was shown that all MS indicators, including incidence and prevalence, have a positive and significant correlation with the Human Development Index. Also, in the study by Liu et al. [23], a strong correlation was reported between the prevalence of MS and SDI. Zhao et al. [25], who focused on adolescents and young adults aged 15–39, also showed that regionally, the standardized disability-adjusted life years (DALYs) have an inverse V-shaped relationship with SDI. In other words, high-income countries have higher reported incidence rates due to better access to diagnostic facilities, stronger disease registration systems, and greater awareness among physicians and the public, while in low-income countries, detecting a significant number of cases is a serious challenge due to the lack of diagnostic facilities and comprehensive registration systems.

Across the six WHO regions, the European region had the highest ASIR in 2021, with an incidence rate of 2.57 per 100,000 population, while the Western Pacific region had the lowest rates at 0.15 and the African region had the lowest rates at 0.31 per 100,000 population. This pattern of geographic distribution is consistent with the hypothesis of a latitude effect on the risk of MS, with regions further from the equator having higher incidence rates [26, 27]. However, recent evidence challenges the primacy of the latitude hypothesis and suggests that healthcare access may be a significant confounder. A global analysis demonstrated that the association between latitude and MS decreased substantially when adjusting for health expenditure, indicating that diagnostic capacity explains a significant portion of the observed latitude effect [28]. In a systematic review by Lane et al. [29], which included 64 articles from 24 countries, it was found that the studies were mainly from Italy, Norway, and Canada, and no studies were found from Africa or South-East Asia. This large knowledge gap in the African and South-East Asian regions, which have the lowest incidence rates in the present study, suggests that the incidence of the disease in these regions could be very significant. For example, in a review by Aderinto et al. [30], the prevalence of MS in Algeria was reported to be 103 per 100,000, which is a significant figure and suggests that in some African countries, the incidence of the disease could be much higher than the average for the African region. However, the same study showed that the African continent faces a severe shortage of diagnostic facilities, including magnetic resonance imaging machines and neurologists, and many African countries have not yet reported any data on MS. Also, in a study by Koh et al. [31] conducted in Singapore, significant differences in disease characteristics were observed between Chinese, Malay, and South Asian ethnic groups, indicating that genetic factors, along with environmental and socioeconomic factors, play a role in the occurrence of this disease.

A persistent gender gap was observed across all regions studied, with incidence rates consistently and substantially higher in women than in men. Globally, the ASIR remained relatively stable in both sexes from 1990 to 2021, with female rates decreasing slightly from 1.02 to 1.01 and male rates decreasing from 0.52 to 0.50 per 100,000 population. The gender gap was most pronounced in EURO (female-to-male ratio: 2.0:1) and High SDI (2.2:1) regions, while it was narrower in AFRO (1.6:1) and Low SDI (1.7:1) regions. This pattern is consistent with previous studies that have shown that the female-to-male ratio in MS can be as high as 2:1 or even higher [32–34]. In Japan, the female-to-male ratio increased from 2.6 in 2001 to 4.0 in 2021 [35]. The cause of the persistent and widespread sex gap in MS is not yet fully understood, but a combination of hormonal factors, X-linked genetics, and immunological differences between the two sexes appear to play a role [36–38].

In analyzing time trends using joinpoint regression, the present study found that the AAPC for the entire period globally was −0.08%, indicating a very mild downward trend. However, examining the trend segments revealed an interesting oscillatory pattern: a significant decrease in 1990–1993 with an APC of −0.66%, then a significant upward trend in 1996–2004 with an APC of 0.36%, and again a decrease in 2004–2012 with an APC of −0.19%, and finally a mild but significant decrease in 2012–2021 with an APC of −0.08%. These fluctuations can be attributed to several factors, including changes in diagnostic criteria, access to magnetic resonance imaging, and improvements in disease registration methods. A systematic review showed that the incidence of MS significantly increased in 38 studies (61%), decreased in 13 studies (21%), and remained stable in 11 studies (18%) [29].

In the present study, the trend patterns in regions with different SDI were very diverse and noteworthy. In the region with a low SDI, the AAPC for the entire period was −0.08%, but in recent years, i.e., the period 2010–2021, a complete reversal of the trend occurred, and the positive APC was estimated at 0.55%, which was statistically significant. In other words, regions that are less economically developed have experienced a significant increase in the incidence of MS in the last decade. In the region with a middle SDI, the AAPC for the entire period was an increase of 0.63%, and in the last period, i.e., the years 2019–2021, the APC reached 1.04%, which is the highest increase among all SDI regions. In the region with an upwardly moderate SDI, the strongest overall upward trend was observed with an AAPC of 0.82%, mainly due to a significant jump with an APC of 1.44% between 1995 and 2003. In contrast, in the region with a high SDI, the average APC for the entire period was −0.03%, indicating a very slight decrease and relative stability, and the trends were mostly fluctuating. At the WHO regional level, the European region showed the highest overall increase (mean APC of 0.70%) and a significant jump between 1996 and 1999 with an APC of 2.18%. This significant jump can be attributed to the introduction of the McDonald diagnostic criteria in 2001 and the widespread availability of magnetic resonance imaging in Europe in the late 1990s [39, 40]. The evolution of MS diagnostic criteria has been closely linked to observed changes in incidence trends across different regions. The introduction of MRI into the McDonald criteria in 2001, followed by revisions in 2005, 2010, 2017, and 2024, progressively increased diagnostic sensitivity and allowed for earlier diagnosis [41, 42]. A nationwide Finnish registry study demonstrated that age-adjusted MS ASIR increased from 3.7 per 100,000 during the Schumacher criteria era (1974–1982) to 9.2 per 100,000 during the early McDonald criteria era (2001–2016), with the sharp rise attributed to MRI incorporation, followed by stabilization to 8.6 per 100,000 under the 2017 criteria [43]. This pattern is consistent with our observed sharp increase in EURO (APC: 2.18% in 1996–1999) and the subsequent stabilization in recent periods, suggesting that diagnostic advances may have contributed to these trends. In addition, real-world data from France showed that the 2017 McDonald criteria reduced median time-to-diagnosis by 3.7 months compared to the 2010 criteria [44]. In contrast, the South-East Asia region showed an overall decrease with an AAPC of −0.12%, but importantly, the APC reached 1.27% between 2019 and 2021, the highest increase of all WHO regions in recent years. This reversal of the trend in the South-East Asia region is a serious alarm for countries in this region and indicates that despite the long-term downward trend, there has been a significant acceleration in the increase in this region in recent years.

The current study’s projections for 2030 showed that globally, the ASIR based on both the APC and AAPC methods will decrease slightly from 0.753 to 0.747 per 100,000, equivalent to a relative decrease of 0.8%. However, regional findings were remarkably diverse. In regions with low-middle SDI, middle SDI, high-middle SDI, as well as in the AMRO, EMRO, EURO, and WPRO regions, the projections indicate increasing trends by 2030. The largest relative increase based on the primary projection method was observed in the SEARO at 12.3%, followed by the low-middle SDI region at 8.2% and the middle SDI region at 9.8%. The study by Liu et al. [23] predicted that the incidence rate would increase gradually until 2050, while mortality and DALYs would decrease.

The back-testing validation in this study provides empirical evidence that the APC method is the preferred projection approach in 10 out of 12 regions (83.3%), while the AAPC method performs better only at the global level and in the EURO region. This finding is supported by methodological literature which highlights that while the estimated annual percentage change (EAPC) offers simplicity in summarizing long-term trends, the AAPC and by extension the APC can more effectively capture nonlinear patterns and recent trend dynamics, though with increased complexity. In regions experiencing rapid epidemiological transitions, short-term trend methods are particularly valuable for detecting recent reversals or accelerations that long-term averages may obscure [45]. The global preference for the AAPC method (MAPE: 0.09% vs. 0.30% for APC) aligns with the stable global ASIR trend documented for MS from 1990 to 2021. A comprehensive analysis of six major immune-mediated inflammatory diseases (IMIDs), including MS, reported declining global trends in ASIRs [46]. This long-term stability—characterized by a gradual and consistent decline—explains why the AAPC, which reflects the overall historical trend, demonstrates superior predictive accuracy at the global level, where recent fluctuations have not substantially deviated from the long-term pattern.

The sensitivity analysis demonstrated that global projections are highly robust, with the global and EMRO regions showing identical projections from both scenarios (sensitivity index = 0). However, substantial uncertainty was observed in SEARO (relative change: 12.0%) and low-middle SDI (8.2%), where trend reversals occurred between primary and alternative scenarios. These findings align with established epidemiological forecasting frameworks, which emphasize that sensitivity analysis identifies regions where improved data collection and refined understanding are most needed [47]. The EURO region showed the largest absolute sensitivity index (0.118), reflecting its high baseline ASIR and divergence between short-term and long-term trends. As noted in GBD forecasting studies, regions with established epidemiological patterns may still show significant absolute sensitivity due to the magnitude of baseline rates [48].

The results of the present study have important implications for health policy. First, the stability of the global ASIR, alongside the 49.9% increase in the absolute number of new cases (from 41,970 in 1990 to 62,920 in 2021), suggests that health systems worldwide need to prepare themselves to manage the growing population of MS patients, driven largely by population growth and aging rather than increased risk. In regions such as SEARO, where the ASIR is projected to increase by 12.3% by 2030, and in low-middle and middle SDI regions, where recent APC values reached +0.93% and +1.04% respectively (2019–2021), there is an urgent need to strengthen diagnostic and treatment infrastructure. These regions are experiencing a significant acceleration in MS ASIR and will require substantial investment in neurological services, MRI accessibility, and disease-modifying therapies to address the growing burden. In a study by Gómez-Figueroa et al. [49], which examined the burden of MS in Mexico, it was shown that the incidence rate is 0.65 per 100,000 population, with significant regional differences in the burden of the disease across states in the country, largely related to socioeconomic factors.

### Study limitations

Limitations of the present study include reliance on the Global Burden of Disease 2021 data, the quality and availability of which vary across countries. Many low- and middle-income countries lack comprehensive MS registries, which could lead to an underestimation of true cases. Also, changes in MS diagnostic criteria over time could have affected the comparability of time trends. This is particularly relevant for regions such as AFRO and SEARO, where MS surveillance infrastructure is less developed. The modeled nature of these estimates introduces uncertainty that may affect the identification of joinpoints and the accuracy of future projections. The extent to which modeled data influenced our inflection points and projections is difficult to quantify, and the projections should be interpreted with appropriate caution for regions with limited primary data. Furthermore, the GBD estimates are subject to revisions in subsequent iterations, and comparisons across different GBD cycles may yield slightly different results.

### Strengths of the study

Using standard methods of the Global Burden of Disease and joinpoint regression, the present study provides a comprehensive picture of global and regional trends in the ASIR of MS and can serve as a basis for more detailed studies in the future. Among the outstanding strengths of this study are the long- and up-to-date time coverage of 32 years (1990–2021), which has enabled the examination of long-term trends and the identification of structural changes in the epidemiology of the disease. In addition, systematic comparisons between regions with different SDI (from low to high) as well as within the six WHO regions significantly increase the power of regional analyses and reveal patterns of health inequalities. The use of the advanced joinpoint regression method allows for the accurate detection of trend breakpoints and the calculation of APC with high accuracy, providing a more realistic understanding of epidemiological turning points than conventional methods. Also, calculating scenario-based sensitivity analysis is another strength of the present study, which provides a more reliable assessment of forecast stability across different regions. Relying on uniform international standards in GBD data also ensures the comparability of findings globally and minimizes errors caused by heterogeneity in diagnostic methods or data recording.

## Conclusions

Overall, this study provides a comprehensive analysis of global and regional trends in MS ASIR from 1990 to 2021 with projections to 2030. The findings reveal that while the global ASIR has remained relatively stable over the past three decades, the absolute number of new cases has substantially increased, reflecting the impact of population growth and aging. Global trends exhibit complex oscillatory patterns, including periods of decline, stability, and increase, likely reflecting changes in diagnostic criteria, the introduction of advanced imaging techniques, improvements in disease registration, and potential environmental or lifestyle factors. Projections indicate that while the global ASIR will continue its mild decline, most regions—particularly Southeast Asia and middle-income regions—are expected to experience increasing ASIRs by 2030. The back-testing validation provided empirical support for method selection, demonstrating that short-term trend methods generally outperform long-term averages in capturing recent epidemiological dynamics. Sensitivity analysis confirmed that global projections are robust, although substantial uncertainty exists in regions with divergent short- and long-term trends, such as Southeast Asia and low-middle SDI regions. These findings have important implications for health policy and resource allocation. The stability of global ASIR alongside increasing case numbers highlights the need for health systems to prepare for a growing population of MS patients requiring long-term care. The rising burden in lower-income and transitioning regions underscores the urgent need to strengthen diagnostic infrastructure, improve access to disease-modifying therapies, and enhance epidemiological surveillance systems. Furthermore, the substantial heterogeneity observed across regions emphasizes that global health strategies must be tailored to local epidemiological contexts rather than applying uniform approaches. The study also highlights critical gaps in MS surveillance, particularly in regions with lower healthcare resources, where accurate case ascertainment remains challenging. Future research should prioritize improving data quality in underrepresented regions, exploring the drivers of recent trend reversals, and developing more refined forecasting models that incorporate emerging risk factors and healthcare interventions. Ongoing validation of projection methodologies is essential to ensure that health policy decisions are informed by the most accurate and context-specific evidence available.

## Data Availability

The data that support the findings of this study are available from the corresponding author upon reasonable request.
